# Treatment of lumbosacral spinal tuberculosis by one-stage anterior debridement and fusion combined with dual screw-rod anterior instrumentation underneath the iliac vessel

**DOI:** 10.1186/s12891-016-0902-5

**Published:** 2016-02-01

**Authors:** Ting Zhang, Xijing He, Haopeng Li, Siyue Xu

**Affiliations:** Department of Orthopaedic Surgery, the Second Affiliated Hospital of Xi’an Jiaotong University, 157 Xiwulu, Xi’an, 710004 Shaanxi P.R China

**Keywords:** Lumbosacral spinal tuberculosis, Anterior debridement, Instrumentation, Screw-rod construct

## Abstract

**Background:**

There has been no consensus regarding what is the optimal means of treating lumbosacral segment tuberculosis. The aim of this study was to evaluate the clinical outcomes of our newly developed one-stage anterior debridement and fusion combined with dual screw-rod construct anterior instrument underneath the iliac vessels for lumbosacral spinal tuberculosis.

**Methods:**

We retrospectively reviewed 22 patients with lumbosacral spinal tuberculosis who underwent one-stage anterior debridement and fusion combined with dual screw-rod anterior instrument underneath the iliac vessels between January 2004 and June 2013. We assessed the visual analogue scale (VAS), erythrocyte sedimentation rates (ESR), neurological performance, kyphotic angles, fusion rates, and computed tomographic angiography (CTA) before and after surgery.

**Results:**

All patients were followed-up for a mean of 46.59 months. There were no instances of spinal tuberculosis recurrence. The mean VAS scores and ESR decreased significantly from the preoperative levels both postoperatively and at the final follow-up (all P <0.001). The mean kyphotic angle significantly increased from the mean preoperative angle both postoperatively and at the final follow-up (both P <0.001). All patients had bone fusion at a mean of five months after surgery. No postoperative vascular complications were observed.

**Conclusions:**

Our findings suggest that anterior radical debridement, fusion combined with dual screw-rod anterior instrument underneath the iliac vessels can be an effective and safe treatment option for lumbosacral segment tuberculosis.

## Background

Lumbosacral spinal tuberculosis accounts for 2–3 % of all cases of spinal tuberculosis [1]. As with other cases of spinal tuberculosis, conservative treatment is often recommended for lumbosacral spinal tuberculosis patients with signs of abscesses, cavities, sequestra, and sinus formation. Surgical interventions are required for patients with progressive neurological functional disturbances, severe kyphosis, massive cold abscesses and those who have no response to conservative treatment [2, 3]. A number of surgical modalities have been performed on patients with spinal tuberculosis including debridement with anterior spinal fusion, anterior spinal fusion plus posterior spinal fusion, posterior spinal fusion alone, and posterior spinal fusion plus anterior spinal fusion [4, 5]. Up to now, reports on the treatment of tuberculosis in the lumbosacral region of the spine remain rare [3, 6–10]. There was still controversy with regard to the optimal surgical approach for the treatment of lumbosacral segment tuberculosis. In clinical practice, special titanium plate or screw-rod system has been available for patients with lumbosacral tuberculosis who are about to undergo the debridement and fusion due to the peculiar anatomical structures of the lumbosacral spine. However, these special internal fixation constructs will undoubtedly increase the economic burden of patients in developing countries. Meanwhile, a previous biomechanics simulation showed that the biomechanical strength of newly special internal fixation constructs is not equal to conventional pedicle screw-rod system [11]. The authors of this study attempted to achieve the one-stage debridement and fusion and meanwhile reduced patient costs with conventional dual screw-rod system.

The aim of this study was to evaluate the clinical outcomes of a newly designed surgical approach of one-stage anterior debridement, fusion, and dual screw-rod anterior construct underneath the iliac vessels for lumbosacral spinal tuberculosis. We found that our newly developed dual screw-rod anterior construct underneath the iliac vessels does not increase the vascular complications.

## Methods

### Patients

This study was approved by the institutional review board of the Second Affiliated Hospital of Xi’an Jiaotong University and conducted in accordance with the Declaration of Helsinki. All subjects gave their written informed consent. From January 2004 to March 2013, twenty-two patients diagnosed with spinal tuberculosis in the lumbosacral region were treated with one-stage anterior debridement, fusion, and anterior screw-rod fixation at our institution. The tuberculosis was defined according to clinical and radiological findings and histopathological examination after surgery. No patient had a previous history of an anterior lumbar procedure. Before surgery, computed tomographic angiography (CTA) or magnetic resonance angiography (MRA) was performed to define the prevertebral vascular anatomy before the anterior lumbosacral procedure [12, 13]. No occlusion, stenosis, atherosclerosis or other vascular pathology was detected at the lumbosacral spinal region. Patients with open tuberculosis or acute miliary tuberculosis, severe vascular sclerosis of main iliac artery, and severe S1 vertebrae damage which cannot tolerate screw placement were excluded.

**Fig. 1:**
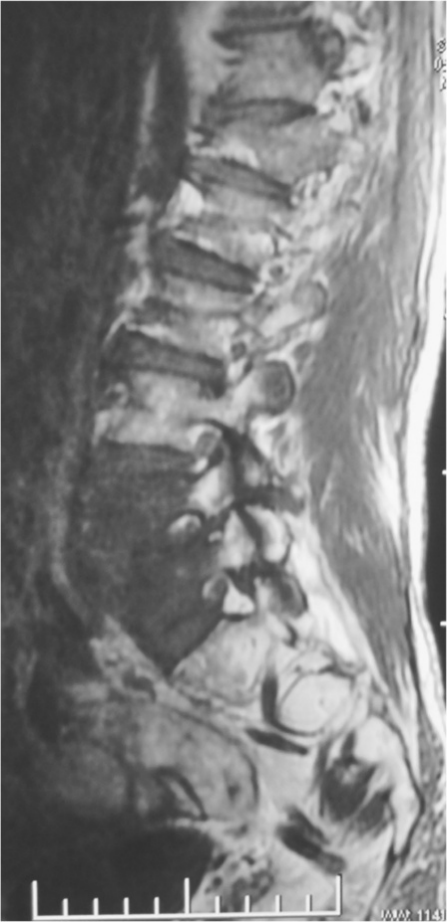
Preoperative magnetic resonance image of a 43 year old male patient with lumbosacral tuberculosis

All the patients exhibited varying degrees of lower back pain. Among them, six patients underwent preoperative lower extremity radicular pain and two patients underwent neurological deficits. Mild to moderate symptoms of tuberculosis were observed in six patients including moderate fever, weight loss, fatigue, and anorexia. Preoperative X-ray, computed tomography (CT) and magnetic resonance imaging (MRI) showed vertebral body damage, intervertebral space narrowing and paravertebral or epidural abscess formation in all patients (Fig. 1).

### Preoperative chemotherapy

We treated all of the patients with standard antituberculosis chemotherapy after the preliminary diagnosis, consisting of oral isoniazid (300 mg daily), rifampin (450 mg daily), ethambutol (1200 mg daily) or pyrazinamide (750 mg daily) and intramuscular streptomycins (0.75 g daily), sustaining for 2–3 weeks before surgery. Surgery was not performed until the toxic symptoms of tuberculosis were controlled. For example, the patients restored to have a normal appetite, presented with the absence of low fever and anemia, and had decreased erythrocyte sedimentation rates (ESR).

### Surgical indications

According to a previous report [14], surgical treatment was needed for patients who had low access to healthcare systems due to low socioeconomic status, documentation of bacterial isolate, previous history of spinal operations, patients who were third-country migrants, prisoners and drug abuser. In our series, most of the patients were from the underdeveloped rural regions of Western China. These patients were patients with poor or without any access to health systems due to low socioeconomic status. The surgical indications of our procedures included persistent severe back pain which have no response to conservative treatment, persistent deterioration of neurologic deficits, severe spine deformity, spinal instability, large abscesses, and/or radiculopathy which have no response to conservative treatment due to compression from the diseased tissue.

### Surgical procedure

**Fig. 2:**
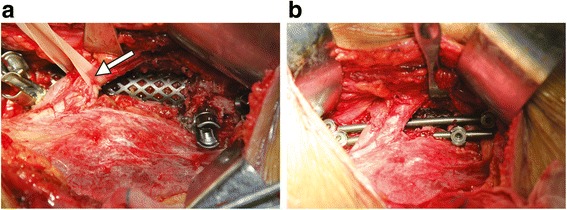
A 43-year-old man with severe low back pain and progressive radicular pain in the lower extremities (Patient 1). **a** Intraoperative photograph showing mobilization of the left common iliac artery and vein (arrow). **b** Intraoperative photograph showing a screw-rod construct underneath the iliac vessel

After induction of general anesthesia with an endotracheal intubation, the patient was placed in a lateral position with the head in a slightly extended position. An oblique flank incision was made to access the lumbosacral spine via an anterolateral retroperitoneal approach. The approach was usually performed on the severely damaged side of the spine. After routine exposure, the infected discs and endplates, caseous necrosis and granulation tissues, sequestrated bone within the vertebral body and the discs above and below the affected vertebral bodies were removed with curettes and laminectomy rongeurs. The paravertebral abscess was then identified and drained. Then, gradual distraction was conducted using intervertebral distraction spacers between the adjacent normal vertebrae to correct kyphosis and restore proper spinal alignment. Thereafter, the spinal defect was measured and repaired with a titanium mesh cage filled with morselized autologous bone at an appropriate length. Subsequently, the anterior instrumentation was performed following autologous iliac bone graft. The components in the newly developed anterior screw-rod construct were all from the classic posterior pedicle screw-rod system (Beijing Fule Science & Technology Development Co., Ltd. Beijing, China), which can reduce the cost of research and development in new construct and spare tuberculosis patient costs. Two screws (diameter: 6.5 mm) were inserted at the normal vertebral body above the affected lesion. The remaining two screws were inserted at S1 vertebrae. The screws were placed in the lateral and anterolateral sides of the vertebral body at an inclination of 45° angle via the anterolateral portal, in a direction towards the contralateral pedicle while keeping away from the spinal canal. Two rods (diameter, 6 mm) were contoured to the normal sagittal profile of the lumbosacral spine. The rods were longitudinally engaged in the screws. The set screws were tightened under compression. For dual screw-rod fixation, a secondary screw-rod construct was applied. A mix of uniaxial and polyaxial screws allowed for easier rod introduction (Fig. 2). Normal saline was utilized for space irrigation to clear the residual tuberculous tissue following careful hemostasis. Then, 1.0 g of streptomycin was administered in the operative region. Thereafter, a drainage tube was inserted before closing the incision. The resected specimens were sent for histopathologic examination. Finally, drainage and incision sutures were performed.

### Postoperative treatment

After surgery, negative suction drain was applied for 48 to 72 hours in all patients. Two weeks after surgery, patients were allowed to start walking with the support of orthosis. Orthosis support was maintained for three months. After surgery, we treated patients with standard antituberculosis chemotherapy regimen for 12–18 months. The usual regimen was oral 300 mg of isoniazid, 450 mg of rifampin, and 1200 mg of ethambutol or 750 mg of pyrazinamide daily. The patients were regularly followed up for assessing hepatic and renal function and ESR.

### Follow-up and medical assessments

**Fig. 3:**
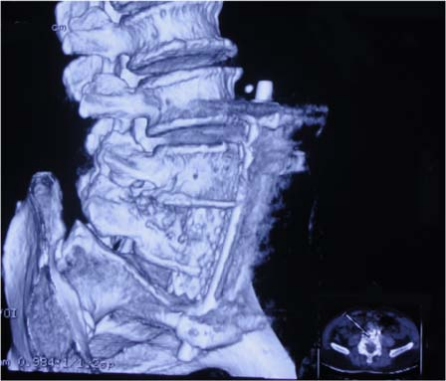
Postoperative three dimensional reconstructive computed tomography (CT) showing the position of titan cage and screw-rod construct

Patients were followed up monthly for the first 3 months, every 3 months until the 12th month, and every 6 months thereafter after treatment. The visual analogue scale (VAS) score, ESR and kyphotic angle were assessed preoperatively and postoperatively. The kyphotic angle was measured by drawing lines along the posterior border of S1 and the posterior border of the first normal vertebra above the affected lesion [15]. CT or anteroposterior and flexion/extension radiographs were obtained to assess the status of bone fusion and if there existed instrumentation failure and/or any recurrence of the disease (Fig. 3). Successful fusion was defined as: 1) presence of trabecular bone bridging between the grafts and the vertebrae; 2) the absence of local pain; and 3) tenderness over the site of fusion [16].

**Fig. 4:**
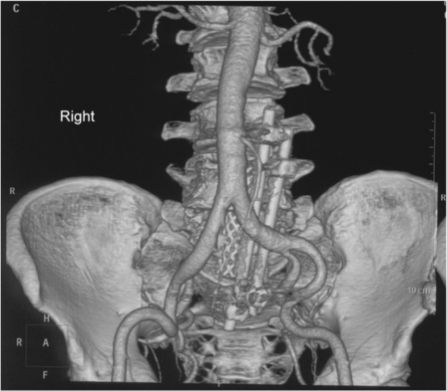
Postoperative computed tomographic angiography (CTA) showing iliac artery adjacent to the lumbosacral spine at the 104 months’ follow up

**Fig. 5:**
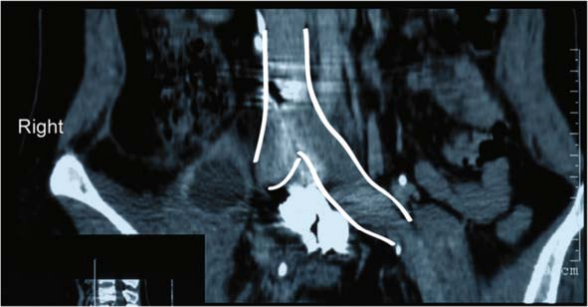
Postoperative computed tomographic angiography (CTA) showing iliac vein adjacent to the lumbosacral spine (outlined by white lines) at the 104 months’ follow up

Postoperative CTA was conducted to assess the integrity of the iliac vessels adjacent to the lumbosacral spine as well as to detect possible iatrogenic vascular injury, stenosis or occlusion (Figs. 4 and 5). The preoperative and postoperative neurologic status and back pain were evaluated using the Frankel Grading System and Visual Analogue Scale (VAS) score, respectively.

### Statistical methods

All analyses were performed by using SPSS statistical software package (version 14.0, SPSS Inc, Chicago, IL, USA). The preoperative, postoperative and final follow-up kyphotic angles, VAS scores, and ESR were compared with repeated measures analysis of variances. A P value of less than 0.05 was considered as statistical significant.

## Results

Table 1 summarizes the patients’ demographics, operative information and disease characteristics. The patients consisted of 13 men and 9 women. The mean age was 41.09 ± 15.24 years (ranged 21–71 years) and the mean duration of symptoms was 9.73 ± 9.52 months (ranged 1–35 months). The lesions were located at the following levels: L4-S1 (5 cases), L5-S1 (5 cases), L4-L5 (11 cases), and L5 (1 case). The mean duration of surgery was 170.5 ± 27.86 minutes, while the mean volume of blood lost during surgery was 694.1 ± 184.7 mL. The mean length of postoperative follow-up was 46.59 ± 19.97 months.

**Table 1:** Patient demographics, operative information and disease characteristics

Patient	Age (years)	Gender	Operation time (min)	Blood loss (mL)	Level	Duration of symptoms (months)	Length of follow-up (months)
1	43	M	170	560	L4-5	24	104
2	68	M	120	500	L4-5	3	24
3	71	M	150	860	L5	15	30
4	36	F	180	600	L4-5	35	44
5	21	M	240	900	L4-S1	2.5	45
6	29	M	200	680	L5-S1	28	60
7	30	F	210	700	L5-S1	2.5	43
8	28	M	150	570	L4-5	6.5	71
9	40	M	130	450	L4-S1	3	39
10	50	M	190	650	L4-5	5	50
11	25	M	170	800	L4-S1	3	46
12	68	F	180	710	L4-5	7	37
13	44	M	160	420	L4-S1	12	82
14	31	F	140	600	L5-S1	4	44
15	32	M	150	1100	L4-5	24	63
16	51	F	170	620	L5-S1	6	55
17	48	M	160	820	L4-5	4	28
18	46	M	190	790	L4-5	8	35
19	21	F	160	920	L4-5	11	46
20	43	F	200	540	L4-S1	5	30
21	23	F	180	480	L5-S1	4.5	24
22	56	F	150	1000	L4-5	1	25
Mean ± SD	41.09 ± 15.24		170.5 ± 27.86	694.1 ± 184.7		9.73 ± 9.52	46.59 ± 19.97

SD, standard deviation; M, male; F, female

**Fig. 6:**
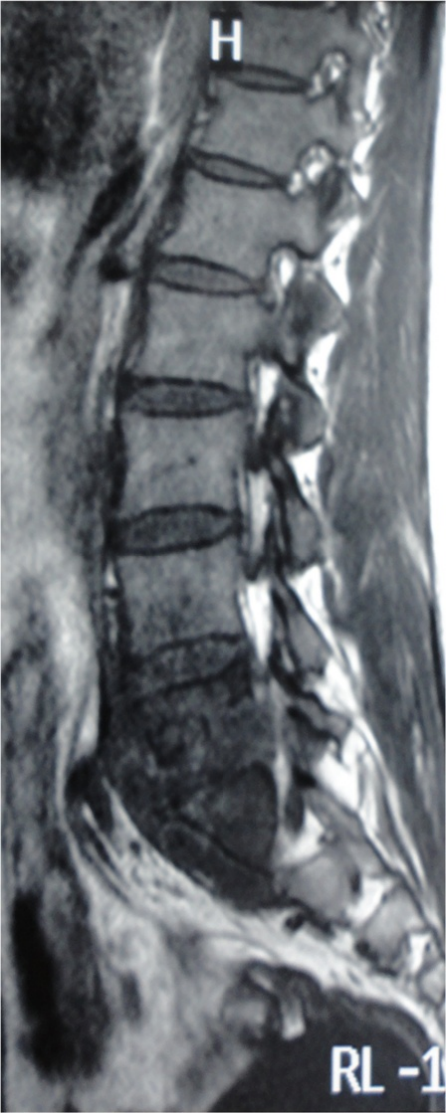
Preoperative magnetic resonance image of a 23 year old female patient with lumbosacral tuberculosis

**Fig. 7:**
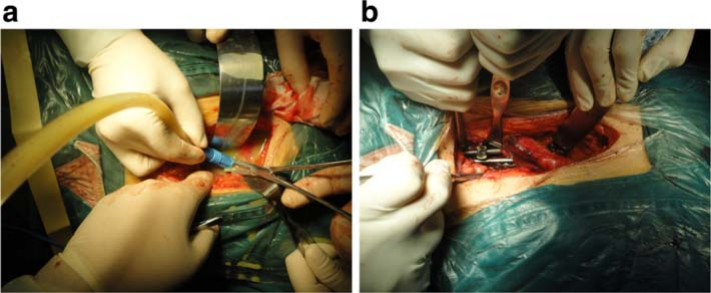
A 23-year-old woman with severe low back pain and right lower quadrant abdominal pain (Patient 21). **a** Intraoperative photograph showing a huge abscess in the paravertebral retroperitoneum. **b** Intraoperative photograph showing a screw-rod construct underneath the right iliac vessel

**Fig. 8:**
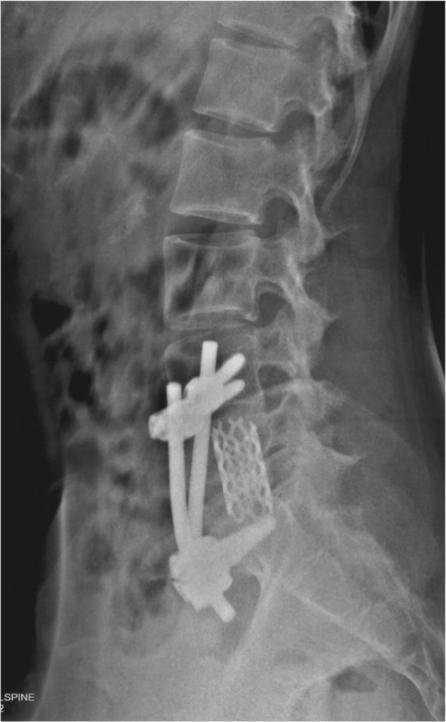
Postoperative lateral radiograph of screw-rod construct fixation underneath the iliac vessels in combination with one-stage anterior debridement and fusion for lumbosacral spinal tuberculosis

**Fig. 9:**
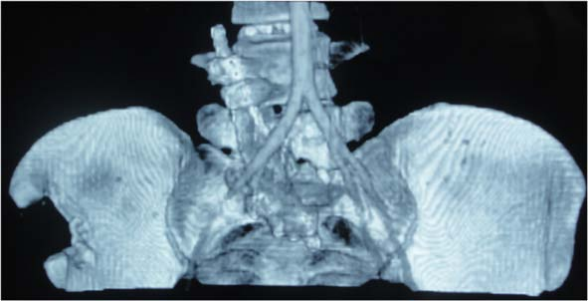
Postoperative computed tomographic angiography (CTA) showing iliac artery adjacent to the lumbosacral spine at the 24 months’ follow up

**Fig. 10:**
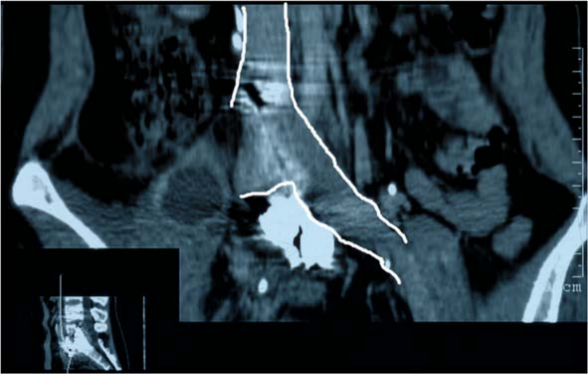
Postoperative computed tomographic angiography (CTA) showing iliac vein adjacent to the lumbosacral spine (outlined by white lines) at the 24 months’ follow up

Table 2 summarizes the radiological and clinical outcomes. There was a significant difference among the preoperative, postoperative and final follow-up with regard to the VAS score (P <0.001) and the ESR (P <0.001). The mean kyphotic angle postoperatively was significantly higher compared to preoperative values (P <0.001). The VAS scores and ESR were all significantly lower at three to six months postoperatively and at the final follow up than preoperatively (all P <0.001). All wounds healed without chronic infection and wound sinus formation. No instrumentation-related complication was noted. Bone fusion was evident at a mean of 5 months (range: 3 to 7 months) after surgery. Figs. 6, 7, 8, 9 and 10 shows preoperative and postoperative radiographs and photographs from one 23 year old female case. No recurrence was observed in any of the patients at the final follow up. None of the patients had disease recurrence at the final follow up and no patients died. Two patients had evidence of neurological impairment before surgery as indicated by Frankel grade D; all other patients had a normal neurological status (i.e., Frankel grade E). After surgery, none of the patients had evidence of neurological impairment.

**Table 2:** Summary of radiological and clinical outcomes

Case	Age (years)	Gender	Kyphotic angle (°)			VAS score			ESR (mm/h)			Bone fusion(months)
			Preop	Postop	Final	Preop	Postop	Final	Preop	Postop	Final	
1	43	M	28.7	43.1	39.5	7	2	0	98	18	12	3
2	68	M	55.1	59.6	58.8	7	1	1	89	15	14	6
3	71	M	40.9	49.8	47.5	8	1	1	60	10	11	4
4	36	F	56.8	62.6	61.0	8	1	1	39	17	13	7
5	21	M	40.0	52.1	49.3	9	2	1	45	15	12	4
6	29	M	37.9	45.5	42.4	5	3	1	36	8	9	5
7	30	F	37.5	44.4	41.1	8	2	0	60	11	10	7
8	28	M	41.8	43.4	43.3	7	1	1	22	10	11	4
9	40	M	17.0	28.0	32.2	6	1	0	44	20	16	6
10	50	M	38.5	50.2	50.6	7	2	1	56	12	7	5
11	25	M	28.8	32.5	33.1	8	2	1	32	10	9	6
12	68	F	36.3	41.1	38.3	8	2	1	50	15	13	7
13	44	M	39.1	42.6	43.2	9	1	1	64	23	19	3
14	31	F	36.7	37.5	37.1	7	3	1	34	18	9	5
15	32	M	31.7	35.6	35.2	6	1	1	35	14	7	6
16	51	F	34.5	35.8	33.4	9	1	1	80	25	20	4
17	48	M	37.4	46.4	44.7	6	2	0	77	17	16	6
18	46	M	29.6	43.7	41.5	9	2	1	67	11	14	4
19	21	F	38.8	47.7	48.2	9	1	1	55	22	18	7
20	43	F	36.9	40.3	39.6	8	2	1	40	14	15	5
21	23	F	37.9	44.1	42.6	6	2	1	43	19	11	5
22	56	F	10.2	19.0	18.1	6	1	1	29	10	5	4
Mean ± SD	41.09 ± 15.24		36 ± 10	42.95 ± 9.62*	41.85 ± 9.17*	7.41 ± 1.22	1.64 ± 0.66*	0.82 ± 0.39*	52.5 ± 20.26	15.18 ± 4.74*	12.32± 3.97*	5.14 ± 1.28

## Discussion

Up to now, there is a lack of consensus regarding the optimal therapeutic modality for treatment of lumbosacral segment tuberculosis. The patient’s unique characteristics, the available resources and the surgeon’s preference are used to determine the optimal method. Anterior radical resection and bone grafting is a very important technique because it allows direct access to the diseased vertebral segments and allows the surgeon to perform radical surgical debridement of the infected tissues [2]. However, stand-alone anterior fusion does not provide sufficient lumbosacral stabilization. Thus supplementary instrumentation is often necessary to improve the fusion rates by increasing the fusion mass stability. Some previous studies have demonstrated that supplementary posterior fixation for lumbosacral segment tuberculosis improves clinical outcomes and fusion rates [7, 17, 18]. However, supplementary posterior instrumentation involves a longer operation time which may lead to substantial blood loss. As a result, one-stage anterior instrumentations have been developed.

Various anteriorly placed, low-profile lumbosacral plates have been developed and applied clinically to match the anatomy of the lumbosacral spine. Beaubien et al. conducted an in vitro biomechanical comparison between anterior lumbar interbody fusions with anteriorly placed lumbar plates, and posteriorly placed pedicle screws or translaminar screws, they concluded that anterior lumbar plating adds significant stability to an anterior lumbar interbody fusion and thus can be considered as a valuable single-approach alternative to supplemental posterior fixation [19]. Gerber et al. conducted a comparative study of the biomechanical differences between anterior lumbar interbody fusion (ALIF) using cylindrical threaded cages alone or combined with an anterior screw-plate or posterior pedicle screws-rods, they found that the anterior screw-plate significantly reduced range of motion and increased stiffness as comparison to stand-alone interbody cages [11]. Similar findings were found in a previous report by He et al. [3].

Zaveri et al. advises against the application of anterior instrumentation at the L4-L5 and L5-S1 segments due to the major vessels crossing the lateral aspects of the vertebral bodies [20]. While some authors suggest that the anterior instrumentation should be applied avoiding the major iliac vessels [3, 8]. Most of the aforementioned anterior lumbosacral plates are only utilized in the anterior position below the bifurcation of the great vessels [2, 3, 11, 19]. To avoid contact with the great vessels, an anterior single screw-rod construct must be placed via the lateral aspect of the lumbosacral vertebral body. The gap, between the lateral aspect of the lumbosacral vertebral body and the anteromedial aspect of the iliac ilium and the great iliac vessels, would potentially accommodate a low profile single screw-rod construct.

Contact between the anterior construct and iliac vessel should be avoided according to the above suggestions. Our newly developed surgical modality (i.e. smooth surface of the screw-rod anterior construct was placed underneath the iliac vessels) can avoid or reduce the increase of the vascular injuries while keeping the contact between the anterior construct and iliac vessel. The components of the newly developed anterior screw-rod construct are all from the classic posterior pedicle screw-rod system. The posterior pedicle screw-rod system was moved to the anterolateral aspect of the lumbosacral spine. Our anterior dual-rod construct needs to cross the vertebral lesion and be placed underneath the iliac vessels. Since the rods pass under the iliac vessels, slightly extensive ligation and mobilization of the iliac vessels required compared to that for autografts alone, especially on the lateral aspects of the vertebral bodies. In this study, we did not observe the incidence of vascular injury after a long-term of follow up.

In this study, some measures were taken to avoid intraoperative and postoperative risk of vascular injury. Preoperative CTA was performed to evaluate the great vessels. A previous report also approved the use of a preoperative CT angiogram to more accurately define the prevertebral vascular anatomy [12]. Anterior surgery is not an option when severe atherosclerotic diseases is detected [12]. In this study, no atherosclerotic pathology was detected at the lumbosacral region in any of our patients. Preoperative evaluation of the prevertebral vascular anatomy helps to the early diagnosis of vascular anomalies as well as the precise anterior mobilization of the vessels and reduces the risks associated with complex vascular anatomy and other prevertebral anatomy.

Baker et al. suggest that the hypogastric paramedian retroperitoneal approach significantly increases the incidence of vascular injury [21]. In this study, we used the anterolateral retroperitoneal (“muscle cutting”) approach instead the hypogastric paramedian retroperitoneal approach to reduce the risk of vascular injury.

During the process of procedure, attention should be paid to manipulate, mobilize, and retract the vasculature at risk and to periodically relax the retractors. Due to the mediolateral position of our anterior dual screw-rod construct is close to that of the interbody, the degree of vascular mobilization did not significantly increase. After safe mobilization and ligation, passing the two rods underneath the deep surface of the great iliac vessels did not increase the risk of intraoperative or postoperative complications.

It should be noted that under constant irritation the vessels may be eroded by the metal construct. A few cases of major vessel injury caused by anterior spinal instrumentation have been reported [22–25]. The pulsation of the aorta against the metal implant is considered to cause progressive aortic wall erosion and ultimately rupture [22–25]. After reviewing the above literatures, we found that the components of the metal implants that caused vessel injury in all of the previously reported cases were the prominent parts of the implants, such as the end of the rod, the corner of the fixation implant, the tip of the vertebral screw, and the head of the screw. The contact of the anterior construct with the major vessels in those cases was actually the contact of the “prominent” parts of the anterior construct with the major vessels. Our newly developed anterior screw-rod construct can be placed underneath the iliac vessels. In this anterior screw-rod construct, contact with the major vessels is strictly limited to the smooth surface of the rods, and vascular contact with the “prominent” parts of the anterior construct is avoided. We did not observe postoperative vascular complications with the application of our anterior screw-rod construct after a mean of 46-month follow up.

CTA can provide less artifacts or distortion of the surrounding anatomy, better depict bony architecture, and allow more anatomical evaluation of the prevertebral structures compared to MRI. CTA is more sensitive than ultrasound in the detection of iliac segment vessels [26]. The present study involved long-term follow-up (24–104 month follow-up) of the patients as well as monitored the vascular status with CTA. During this process, we did not observe any occurrence of short-term or long-term vascular injury.

## Conclusions

Anterior radical debridement, fusion, and dual screw-rod anterior construct underneath the iliac vessels can be an effective and safe treatment option for lumbosacral segment tuberculosis. Further studies with a larger number of patients are warranted.

## Abbreviations

ALIF: Anterior lumbar interbody fusion
CT: Computed tomography
CTA: Computed tomographic angiography
ESR: Erythrocyte sedimentation rates
MRA: Magnetic resonance angiography
MRI: Magnetic resonance imaging
VAS: Visual analogue scale
